# Using a *Caenorhabditis elegans* Parkinson’s Disease Model to Assess Disease Progression and Therapy Efficiency

**DOI:** 10.3390/ph15050512

**Published:** 2022-04-22

**Authors:** Samantha Hughes, Maritza van Dop, Nikki Kolsters, David van de Klashorst, Anastasia Pogosova, Anouk M. Rijs

**Affiliations:** 1 HAN BioCentre, HAN University of Applied Sciences, Laan van Scheut 2, 6525 EM Nijmegen, The Netherlands; maritza.vandop@han.nl (M.v.D.); nikkikolsters@live.com (N.K.); david.vandeklashorst@han.nl (D.v.d.K.); anastasia.s.pogosova@gmail.com (A.P.); 2 A-LIFE Amsterdam Institute for Life and Environment, Section Environmental Health and Toxicology, Vrije Univeristeit Amsterdam, De Boelelaan 1085, 1081 HV Amsterdam, The Netherlands; 3 Division of BioAnalytical Chemistry, AIMMS Amsterdam Institute of Molecular and Life Sciences, Vrije Univeristeit Amsterdam, De Boelelaan 1085, 1081 HV Amsterdam, The Netherlands

**Keywords:** *C. elegans*, α-synuclein aggregation, lifespan, mobility, screening platform, therapeutics, Ambroxol, Levodopa

## Abstract

Despite Parkinson’s Disease (PD) being the second most common neurodegenerative disease, treatment options are limited. Consequently, there is an urgent need to identify and screen new therapeutic compounds that slow or reverse the pathology of PD. Unfortunately, few new therapeutics are being produced, partly due to the low throughput and/or poor predictability of the currently used model organisms and in vivo screening methods. Our objective was to develop a simple and affordable platform for drug screening utilizing the nematode *Caenorhabditis elegans*. The effect of Levodopa, the “Gold standard” of PD treatment, was explored in nematodes expressing the disease-causing α-synuclein protein. We focused on two key hallmarks of PD: plaque formation and mobility. Exposure to Levodopa ameliorated the mobility defect in *C. elegans*, similar to people living with PD who take the drug. Further, long-term Levodopa exposure was not detrimental to lifespan. This *C. elegans*-based method was used to screen a selection of small-molecule drugs for an impact on α-synuclein aggregation and mobility, identifying several promising compounds worthy of further investigation, most notably Ambroxol. The simple methodology means it can be adopted in many labs to pre-screen candidate compounds for a positive impact on disease progression.

## 1. Introduction

Parkinson’s Disease (PD) is the second-most prevalent and fastest growing neurodegenerative disease, with 12 million people worldwide expected to be living with PD by 2040 [1,2]. PD is characterized by the accumulation of α-synuclein in the Lewy Bodies, which results in the loss of dopaminergic neurons in the brain [3]. In its pathogenic form, α-synuclein aggregates into the amyloid fibrils, which cause toxicity. Aggregation of α-synuclein is a stochastic event that increases with age, but the rate of aggregation can be accelerated by many additional factors, including the concentration and type of metal ions [4,5], a low pH [6,7], and interactions with lipids [8,9].

Unfortunately, treatment options for people living with PD are very limited, with the most effective therapeutic agent being Levodopa [10,11,12,13,14]. Levodopa acts to replenish dopamine and thus to reduce and delay disease symptoms [15,16], and so it is used in managing symptoms. Targeting the accumulation and aggregation of α-synuclein with a view to slow down, or halt, disease progression is a key goal of pharmacological research [17,18].

To identify new therapeutic compounds that slow disease progression and treat the symptoms, it is preferable to screen for candidates in a high-throughput and accurate manner. The nematode *Caenorhabditis elegans* is ideally suited to such whole-organism screens as it combines the simplicity of cell culture with the complexity of a multi-cellular organism, without the ethical considerations applied to vertebrate animals. *C. elegans* has a short lifecycle and reproduction time, and a high degree of similarity to humans at the genetic [19,20] and cellular [21] levels. The simple anatomy of *C. elegans* consists of tissues and organs also found in humans, including a pharynx, intestine, cuticle, and nervous system. With a completely mapped neural network and connectome [22], the nematode worm is frequently used in neurobiology studies. Hallmarks of neuropathologies have been identified in the nematode [23,24,25], including those for Parkinson’s Disease [26,27,28,29,30], where there are nematodes that recapitulate the most frequent genetic causes of PD, including LRRK2, PARK2, and DJ-1 (reviewed in Maulik et al. [31] and Cooper and van Raamsdonk [32]), as well as modeling α-synuclein expression [27,33,34].

The nematode plays an important role in the drug discovery pipeline, testing the toxicity and efficacy of new pharmaceuticals [35], and has been used to identify inhibitors of α-synuclein aggregation [27,36,37,38,39]. Behavioral phenotypes associated with PD can also be observed in nematodes and used as a readout to gain insight into disease pathology, as well as to identify small-molecule compounds that modify disease. In both cases, automation and machine learning aid efficient high-throughput screening [40,41]; however, this requires specialized knowledge or equipment. Many studies have observed changes in the number, size, or intensity of the α-synuclein aggregations following compound exposure as a readout of potential disease modifiers [39,42,43,44,45], and each method has its advantages and disadvantages. Measuring the fluorescent intensity of the aggregations is prone to artifacts, while on the other hand, arbitrary choices must be made when counting the number of α-synuclein foci [46], and many use techniques that are not simple or require additional training. To overcome these hurdles, we propose an economical and straightforward whole-system approach to screen drugs with the potential to treat PD, using a standard microscope and freely available software with *C. elegans*.

By utilizing the nematode model for PD that over-expresses the human disease-causing α-synuclein fused to a fluorescent reporter in the body wall muscles, strain *NL5901* [27], it is possible to directly monitor α-synuclein aggregation throughout life. This nematode strain mimics the α-synuclein aggregation found in Lewy bodies and idiopathic PD [3,47]; however, it should be noted that this strain does not recapitulate the progressive loss of dopaminergic neurons [48], which is beyond the scope of this work. The open-source ImageJ software [49] was employed to quantify changes in the aggregation of the α-synuclein throughout the lifespan of the nematode in parallel to a behavioral assay as a robust and representative readout of neuromuscular health. Once the parameters were identified, we explored the effect of the most common PD treatment, Levodopa, on the nematodes, to confirm the use of this strain of *C. elegans* for a drug screening platform.

Subsequently, we demonstrated a simple and economically viable platform to screen a variety of small-molecule modulators for PD in an efficient manner, where both the symptoms as well as the cause of the disease were monitored. Repositioning drugs has shown promise for PD treatments [50], with Ambroxol being one such example [51,52]. Ambroxol treatment reduces α-synuclein levels in vitro and in vivo [53,54,55,56] and has proceeded to the clinical trial phase [57,58]. To test the applicability of the *C. elegans* platform, small-molecule modulators used in the treatment of Alzheimer’s and Huntington’s Disease were tested. We confirmed that several of these modulators were able to reduce the α-synuclein aggregation, including Ambroxol, and recover the movement defect in the worm model of PD, demonstrating this economical and simple methodology to screen candidate compounds for a positive impact on disease progression.

## 2. Results

### 2.1. Synuclein Plaques Accumulate as Worms Age, Affecting Mobility but Not Overall Lifespan

The constitutive expression of α-synuclein fused to a yellow fluorescent protein (strain *NL5901*) enables visualization of the accumulation of α-synuclein aggregates throughout the lifespan of the worm [27]. While we were able to confirm the presence of aggregates in the whole animal, we chose to focus on the changes in α-synuclein aggregates in the most anterior region, the head. The head was chosen as this is the region that could be most easily visualized throughout life and used for comparison across ages. The differences in α-synuclein aggregation were observed at three key life stages: L4, 1 week (L4 + 7 days), and 2 weeks (L4 + 14 days) of age.

**Figure 1:**
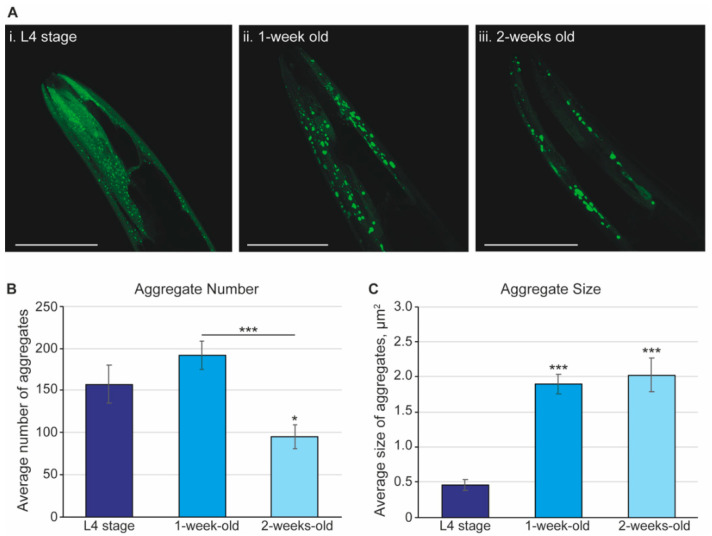
The α-synuclein aggregates display dynamic changes in size and number across the lifespan of *C. elegans*. (**A**) Representative images of the α-synuclein aggregates. Images are maximal projections from a series of 1 µm Z-stacks taken using a Zeiss Axio LSM 700 from worms at L4 (**i**), 1 week, L4 + 7 days (**ii**), and 2 weeks, L4 + 14 days (**iii**) of age. Images are of independent animals and representative of an *n* ≥ 14 over a series of 3 independent experiments. The worm strain is *NL5901* where α-synuclein is fused to a fluorescent reporter in the body wall muscle. Scale bar, 50 µm. (**B**) Quantification of the number of aggregates and (**C**) size of aggregates. Maximal projections of confocal images were quantified using ImageJ with the number and size (in µm^2^) of the aggregates assessed. The dark blue bars show the L4 stage (*n* = 15), the blue bars show 1-week-old animals (L4 + 7 days; *n* = 18), and the light blue bars show 2-week-old animals (L4 + 14 days; *n* = 14). Graphs shown are averages with the standard error of the mean (s.e.m.) from 3 independent experiments. Statistical analysis used the 2-tailed 2-sample *t*-test, where the averages are compared to L4-stage worms, unless otherwise indicated. Asterisks indicate *p*-value, where * *p* < 0.05 and *** *p* < 0.001.

Using confocal microscopy, Z-stacks were taken through the head of each animal and the resulting maximal projections were processed using ImageJ software. Representative images are shown in Figure 1A of L4, 1-week-old, and 2-week-old nematodes. Worms at the L4 stage had a large number of small fluorescent foci, the α-synuclein aggregates. Quantification of these foci over a population of worms indicate that L4 worms have an average of 157 aggregates that are 0.46 µm^2^ in size (Figure 1B,C). There was a large range in the number of plaques present at the L4 stage, confirming observations by others [27,59]. Moreover, the L4 worms displayed background fluorescence, which together suggests that the L4 stage is not the ideal stage to probe α-synuclein aggregation. However, including the L4 stage and applying deconvolution to the image could provide insights into the full aggregation process, but this is beyond the aim and scope of this work. At 1 week of age (L4 + 7 days), worms display an increase in the number and a significant increase in the size of aggregates, from 0.46 µm^2^ to 1.89 µm^2^. In 2-week-old (L4 + 14 days) nematodes, the average size of the α-synuclein aggregates is 2.03 µm^2^, but the number of aggregates is significantly decreased compared to earlier life stages. Our observations are in line with the hypothesis that the process of aggregation is dynamic, whereby the smaller aggregates formed during the aging, i.e., from L4 to L4 + 7 days, can act as “seeds” from which the aggregates grow and fuse together, forming the observed large aggregates [60,61]. The full range, and especially the smaller species, can be an interesting target for disease-modifying therapies [62,63]. As the accumulation of the aggregates is visible, this provides a useful tool to assess the effectiveness of potential drugs.

**Figure 2:**
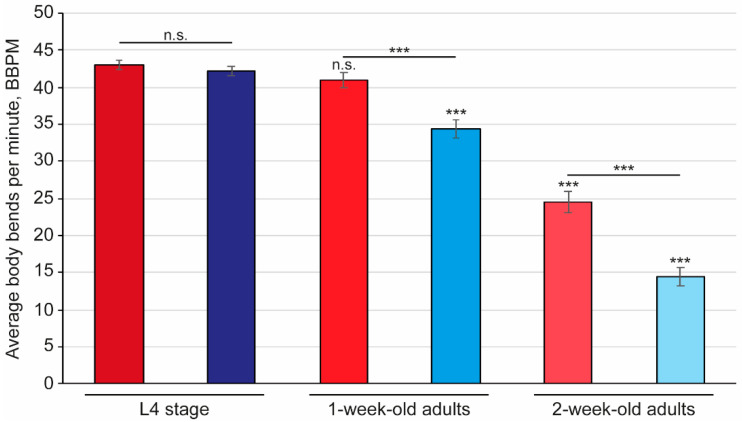
The number of body bends reduced with worm age, with a more pronounced effect in the worm model for Parkinson’s Disease. The number of body bends per minute were assessed in wild-type (red bars; strain, *N2*) and worms expressing the human α-synuclein in body wall muscle (blue bars; strain, *NL5901*) animals at L4, 1 week (L4 + 7 days), and 2 weeks (L4 + 14 days) of age. Counts were assessed using wRMTrck in ImageJ and averages plotted with the standard error of the mean (s.e.m.). Statistical analysis used the 2-tailed 2-sample *t*-test where *** indicates a *p* value of *p* < 0.001 when comparing the same strain to L4, or as indicated, and n.s. designates not significant. *n* ≥ 60 over 3 independent replicates.

Sarcopenia, the progressive loss of muscle mass and function [64,65], is prevalent in older adults living with PD and is associated with the severity of the disease [66,67]. In *C. elegans*, sarcopenia corresponds to a reduction in mobility, which is assessed by counting the number of body bends, or thrashes, in liquid media. A bend was counted as a single head-to-tail touch using an ImageJ plugin (see Supplemental Movie S1). To explore the link between sarcopenia and disease progression, wild-type worms and those that express the human disease-causing α-synuclein were assessed for differences in body bending (Figure 2). At the L4 stage, both wild-type and the nematode model for PD display a similar thrash rate, with an average of 43 body bends per minute (BBPM). As the worms age, their mobility declines, which is clearly observed by the reduction in thrash rate of 5% and 43% after 1 week and 2 weeks, respectively. Interestingly, the worms that express α-synuclein (strain, *NL5901*) display a more rapid decline in mobility, with an 18% reduction in body bends at 1 week of age, and a 65% reduction in body bends at 2 weeks of age.

**Figure 3:**
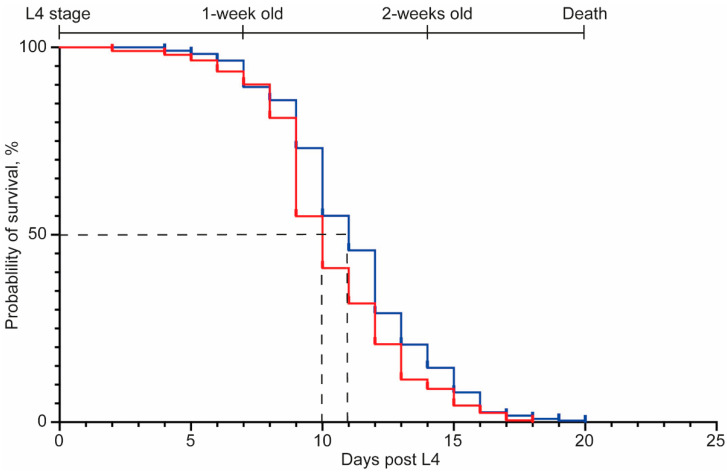
*C. elegans* that express the human α-synuclein have no difference in lifespan compared to control worms. Wild-type animals (strain, *N2*; red line) and those that express the human α-synuclein (strain, *NL5901*; blue line) were observed for survival on NGM in the presence of FUdR, a compound that prevents offspring from hatching. The life stages of nematodes are indicated along the top of the graph and the 50% survival point highlighted by a dashed line. Details of the mean and maximal lifespan are shown in Supplementary Table S1.

As the nematode model for PD had an enhanced age-related reduction in mobility, similar to people living with the disease, we sought to examine if this was reflected in lifespan differences. The maximal lifespan was similar for the wild-type worms as for the worms with the α-synuclein construct (strain, *NL5901*), as was the day at which 50% mortality was observed (Figure 3, Supplementary Table S1). These data suggest that the presence of the α-synuclein aggregates does not impact lifespan.

### 2.2. An Affordable and Simple Platform to Assess Disease Progression and Therapy Efficiency

To gain a detailed understanding of α-synuclein aggregation, a confocal microscope was used. While this provides an accurate assessment of the α-synuclein aggregates, it is a time-consuming and labor-intensive process, as well as being relatively expensive to purchase. Most laboratories have access to a conventional fluorescent microscope, which has the added benefit of being relatively simple to use. We therefore examined whether similar results could be obtained using conventional fluorescent microscopy, in our case, a Zeiss Imager.M2 microscope and Zeiss Axio LSM700, respectively. Images were obtained of L4 and 1-week-old (L4 + 7 days) nematodes on both systems and analyzed with ImageJ. It was not possible to adequately quantify the α-synuclein aggregation in the images of L4 animals generated with the conventional microscope, due to elevated levels of background fluorescence obstructing the actual aggregates. However, the images of 1-week-old nematodes generated using the conventional microscope could be quantified, as they have an increase in the size of aggregates compared to the L4 stage (Supplemental Figure S1). It is challenging to directly compare results, as images from the conventional microscope can only be obtained from one focal plane, while a confocal takes images through the whole thickness of the worm. However, our results demonstrate that by using a conventional fluorescent microscope as found in most laboratories, it is possible to screen worms for changes in the α-synuclein aggregation more quickly than on a confocal microscope. The use of ImageJ to quantify the aggregates is also simple and robust for any operator to use. For this reason, all further experiments were undertaken using a conventional fluorescent microscope, the Zeiss Imager.M2 microscope.

To explore the effect of possible small-molecule inhibitors on the aggregation and mobility of *C. elegans*, we chose to assess worms at 1 week of age (L4 + 7 days). The reason for this was three-fold; first, animals at 1 week of age are best and more reliable to observe and quantify the changes in fluorescently tagged α-synuclein and body bends; secondly, L4 + 7 days showed the best balance of the number of live and dead worms; and thirdly, worms at this stage of development are predicted to be the age of worm that best represents humans with PD [37,68,69].

### 2.3. Levodopa Exposure Recovers the Mobility Defect in a C. elegans PD Model, While the α-Synuclein Aggregates Are Not Affected

**Figure 4:**
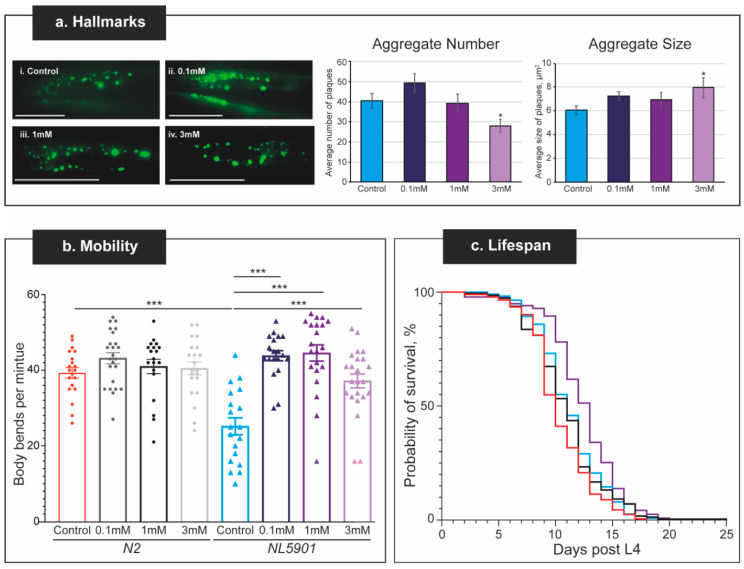
The dose–response curve for Levodopa on thrashing and α-synuclein aggregates. (**a**) Hallmarks of the Parkinson’s Disease shown by the α-synuclein aggregates. Representative Zeiss image of the head of 1-week-old (L4 + 7 days) animal. Representative images of the *NL5901* strain under (**i**) control conditions where the NGM contains FUdR, (**ii**) 0.1 mM, (**iii**) 1 mM, or (**iv**) 3 mM Levodopa. Scale bar, 50 µm. Quantification of the number and size of α-synuclein aggregates from Zeiss images. In both cases, the average is plotted with the standard error of the mean (s.e.m.) with the FUdR control (blue bars; *n* = 62) and 1 mM Levodopa (purple bars; *n* = 24). There is no significant difference in number or size of the aggregates following exposure to 0.1 nor 1 mM Levodopa, but a significant increase in aggregate size (shown by * where *p* < 0.05) at 3 mM Levodopa. (**b**) The average body bends per minute (BBPM) were assessed in either *N2* (circles) or *NL5901* (triangles) nematode strains in control (NGM supplemented with 100 µM FUdR) or Levodopa-supplemented NGM where *n* ≥ 20 for all conditions. BBPM was assessed using wRMTrck in ImageJ and averages plotted with the standard error of the mean in GraphPad Prism v9. Each point represents a single worm assessed. The only statistical difference is comparing *N2* and *NL5901* in the absence of drugs, where the thrashing in *NL5901* is significantly reduced. Asterisks *** indicate a *p* value of <0.001. (**c**) Worms were assayed for survival in the presence of FUdR (wild-type stain, *N2*, red line; PD model strain *NL5901*, blue line) and 1 mM Levodopa (wild-type stain, *N2*, black line; PD model strain *NL5901*, purple line). *C. elegans* that express the human α-synuclein do not show a significant difference in lifespan compared to wild-type worms. Details of the mean and maximal lifespan are shown in Supplementary Table S1.

To test the applicability of our analysis platform to probe drug effectivity, Levodopa was chosen as it is the “Gold standard” treatment for Parkinson’s Disease [70,71]. Concentrations of Levodopa (0.1, 1, and 3 mM) were selected based on the literature [39,72,73] and tested for an effect on α-synuclein aggregation and body bending. There was no significant change in the number nor size of α-synuclein aggregates compared to control conditions at 0.1 mM and 1 mM Levodopa (Figure 4a). However, continuous exposure to 3 mM Levodopa from L4 for 1 week (L4 + 7 days) resulted in a small but significant (*p* < 0.05) decrease in aggregate number but a corresponding increase in their size. There was no effect of the drug on the mobility of wild-type *C. elegans* (Figure 4b), but, in contrast, exposure to Levodopa at all concentrations recovered the body bending in the worms with α-synuclein aggregates. The body bends in the *NL5901* animals at 1 week of age was 25 BBPM, which was increased to 44 BBPM following exposure to 0.1 and 1 mM Levodopa for 1 week, with 3 mM Levodopa also causing the thrashes to increase to 37 BBPM. Together, the recovery in body bending in the *NL5901* nematodes exposed to all concentrations of Levodopa was not significantly different to the body bends of wild-type animals at the same age (Figure 4b), thus demonstrating that Levodopa is able to recover the mobility defect on the worms expressing the human disease-causing α-synuclein.

To assess the effect of Levodopa on lifespan, both wild-type and *NL5901* worms were continually exposed to the drug at 1 mM Levodopa from L4 onwards. This concentration was selected based on the mobility and plaque data, as well as data from the literature [72]. When exposed to Levodopa, wild-type worms have a maximal lifespan of 25 days, while the *NL5901* worms (the nematode PD model) have a maximal lifespan of 20 days (Figure 4c, Supplementary Table S1). While this is significantly different, it is likely to be due to a single wild-type animal. It is of interest to note that the age at 50% mortality is greater in *NL5901* worms, at 13 days compared to 11 days for the wild-type worms exposed to Levodopa.

Our data on Levodopa treatment in *C. elegans* that express the human disease-causing α-synuclein are in line with the effect of Levodopa in humans living with Parkinson’s Disease [12,74,75]. In this way, we have demonstrated that our platform is suitable to screen compounds for a therapeutic effect. The advantage of our platform is that it can be easily applied in a small laboratory setting to screen compounds for a positive effect on PD. To further test the applicability for this, we chose to screen a selection of compounds that may confer a positive effect on α-synuclein aggregation and body bending.

### 2.4. Screening of Small-Molecule Modulators for a Positive Effect on the Hallmarks of PD in C. elegans

The small-molecule modulators that were selected in this study are used to treat PD and other neurodegenerative diseases. Ambroxol [58,76] and Betulin [44,77] were selected as these are treatments for PD. Valproic acid [78,79], Bexarotene [80,81], and Galantamine [82,83,84] were included as potential therapies for people living with PD but are more commonly used to treat Alzheimer’s Disease. Lastly, we tested Tetrabenazine, which is predominantly a treatment for Huntington’s Disease [85,86] but has shown promise for PD patients who have Levodopa-induced dyskinesias [87].

**Figure 5:**
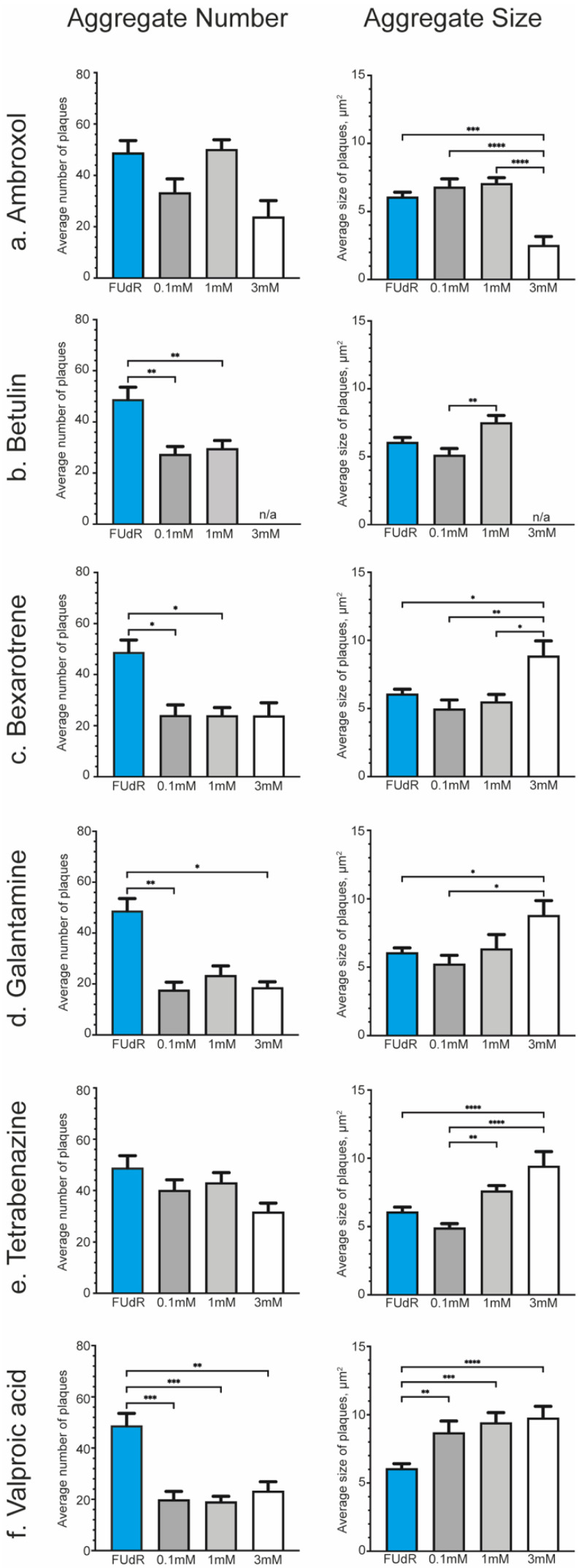
A survey of the effect of drugs on α-synuclein aggregates. Quantification of the number (**left** panel) and size (**right** panel) of α-synuclein aggregates from Zeiss images after exposure to drugs for 1 week (L4 + 7 days) for each drug. In all cases, the average is plotted with the standard error of the mean (s.e.m.). The statistical tests are a one-way ANOVA where * *p* < 0.05, ** *p* < 0.01, *** *p* < 0.005, and **** *p* < 0.001. The blue bars show the control, FUdR, *n* = 62 over 6 independent replicates. All dark grey bars represent exposure to 0.1 mM for 1 week, light grey indicates 1 mM exposure, and white bars are for worms exposed to 3 mM of the drug. (**a**) Exposure to Ambroxol (*n* = 20, 20, 12). Using ANOVA, there was no significant difference between the aggregate number of Ambroxol-exposed worms compared to the control. However, a 2-tailed 2-sample *t*-test gave a *p* = 0.029 between FUdR and 3 mM Ambroxol-exposed worms; (**b**) Betulin (*n* = 33, 36). Note that 3 mM was not tested, as this was found to be toxic to *C. elegans* by other groups; (**c**) Bexarotene (*n* = 20, 22, 21, 15); (**d**) Galantamine (*n* = 13, 7, 11); (**e**) Tetrabenazine (*n* = 22, 22, 15) showed no significant difference between the FUdR control and Valproic Acid-exposed worms nor did a *t*-test; (**f**) Valproic Acid (*n* = 23, 22, 22).

In all cases, worms were exposed to compounds for 1 week (L4 + 7 days) before being used for quantification of the α-synuclein aggregation (Figure 5) and analysis of body bending (Table 1 and Supplemental Figure S2). Lifespan was only evaluated for Ambroxol (Supplemental Figure S3). As, for both Levodopa and Ambroxol, lifespan was not altered (shortened or lengthened), we excluded this for the other small-molecule modulators in this study. However, it can be an interesting hallmark to explore for future lead compounds.

Exposure to Ambroxol showed a reduction in the α-synuclein aggregates (Figure 5a), which were almost completely ameliorated when worms were exposed to 3 mM of the drug (Supplemental Figure S4). While there was no observable recovery of the body bending at 0.1 and 1 mM (Table 1, Supplemental Figure S2), those worms exposed to 3 mM Ambroxol were paralyzed directly upon being placed in the M9 buffer for the mobility assay. It is possible that the mobility defects are a consequence of the elevated solvent percentage [88,89], which, in this case, is 3% DMSO, due to the limited solubility of Ambroxol in lower DMSO concentrations. Worms exposed to 3% DMSO did not display the same level of paralysis nor such a reduction in the α-synuclein aggregates (Supplemental Figure S5). Similarly, we were unable to test Betulin at 3 mM due to its toxicity [44], but at lower concentrations, there was a significant reduction in the number of aggregates (Figure 5b). It was of significant interest to see that exposure to Betulin also resulted in a recovered mobility of the worms that expressed the α-synuclein to comparable levels in the wild-type worms (Table 1, Supplemental Figure S2). Bexarotene at all concentrations significantly reduced the number of aggregates, although at 3 mM, the present aggregates were also significantly larger (Figure 5c). Accordingly, there was a recovery in the mobility defect of this nematode PD model (Table 1, Supplemental Figure S2). Exposure to Galantamine had a similar effect to Bexarotene, but there was no recovery in the mobility of the animals (Figure 5d, Table 1 and Supplemental Figure S2). Exposure to 0.1 mM of Tetrabenazine had no effect on α-synuclein size nor number (Figure 5e). However, higher concentrations of Tetrabenazine did result in a significant increase in aggregate size, while their number remained constant (Figure 5e). There was no recovery in the body bending of *NL5901* worms when exposed to Tetrabenazine, although the 3 mM concentration could not be tested effectively, as the worms were paralyzed when placed in M9 buffer (Table 1, Supplemental Figure S2). Valproic acid was a very interesting compound in that at all concentrations tested, it caused the aggregates to be smaller in number, but with an increase in size. Coincidently, there was a recovery of the mobility of the animals (Figure 5f, Table 1 and Supplemental Figure S2).

Taken together, these data show that the *C. elegans* platform is a robust method to observe changes in the size and number of α-synuclein aggregates, as well as mobility, with a view implicating lead compounds for further, detailed analysis.

## 3. Discussion

*C. elegans* are utilized in phenotypic drug screening as they are small, have high genetic homology with humans, and raise no ethical concerns [90]. While *C. elegans* are used in screens to identify pharmaceuticals for Parkinson’s Disease, many protocols use techniques that require specialist knowledge and/or equipment. We wished to develop an accessible and affordable approach to test the ability of pharmaceuticals to reduce the symptoms and hallmarks of Parkinson’s Disease. A multi-phenotype platform is especially important, as currently no therapeutics exist that are capable of addressing both the symptoms (mobility defects) and cause of the disease (α-synuclein aggregation).

This study has provided additional information into the phenotypic effects of the Parkinson’s Disease model *C. elegans* (strain, *NL5901*). The accumulation of α-synuclein was assessed during the lifetime of the nematode in an affordable, simple, and robust manner. While previous groups have explored the change in the number of α-synuclein aggregates in aging worms, these investigations tend to focus on a shorter period of time, such as for 4 days post-L4 [27,59], despite younger worms being less representative of human PD in an aging population. We confirm that there is no correlation between the presence of aggregates and maximal lifespan, in line with other studies [59,91]. Further, it should also be noted that the disease pathology in *C. elegans* also arises from the fact that normal cellular proteins are more prone to aggregation due to the decline in proteostasis mechanisms [92,93]. These findings in *C. elegans* are parallel to observations in humans whereby the increase in protein aggregation over time corresponds to functional decline [92,94].

To test if this platform assesses potential small-molecule compounds that could interfere with the aggregation process, worms were continuously exposed to the small-molecule drugs from L4 until the drug effect was probed in 1-week-old (L4 + 7 days) worms. Initially, the platform was tested using Levodopa, the “Gold Standard” treatment for PD [70,71]. While Levodopa had no effect on the α-synuclein aggregations, there was a significant recovery of the body bending defect. These results are in line with those from other nematode studies and in mammalian experiments [12,13,39,72] and are analogous to results in humans with PD that take Levodopa [12,74,75]. The strain used here has α-synuclein being expressed in the body wall muscles and, therefore, it raises the question as to how exposure to Levodopa would recover the mobility defect in muscles that are not directly innervated by dopaminergic neurons. In *C. elegans*, dopamine receptors can be found throughout the nematode nervous system, and a connection has been shown between dopamine, motor circuitry, and behaviors including locomotion [95,96,97]. Indeed, two key dopamine receptors, D1- and D2-like receptors (DOP-1 and DOP-3, respectively in *C. elegans*), are present in cholinergic and GABAergic motor neurons, which are key in the control of worm mobility [72,97]. In worms that express α-synuclein, D1 receptor (D1R) expression was increased when worms were exposed to L-DOPA [72], in agreement with findings in mammals [98,99]. Exogeneous dopamine has been shown to control motor function in somatic motor neurons [100], and the depletion of dopamine in people with PD results in the motor symptoms associated with PD. It is therefore possible that the exogeneous dopamine provided to *C. elegans* in the form of Levodopa is able to stimulate muscles and result in an increase in the thrashing observed in the worms that express α-synuclein, while wild-type worms that do not have the integrated transgene do not show such an effect. While this is of great interest and worthy of further investigation, it is currently beyond the scope of this work. However, it does show the relevance of the *C. elegans* model for screening for treatments for PD.

There was no detrimental effect of long-term Levodopa exposure on the overall health and lifespan of either wild-type or PD nematodes. Indeed, Levodopa has a marginal positive effect on health span in PD worms, which may relate to an enhanced quality of life in people living with PD. This is of note, as the age of onset of Levodopa treatment may affect the outcome for the patient in terms of quality of life [101] and the duration of treatment may be of importance to prevent Levodopa-induced dyskinesia [102]. These findings demonstrated that our platform is a suitable method by which to test the effectiveness of drugs on both the symptom (mobility) and cause (α-synuclein aggregation) of PD. This will provide a sound basis from which to move target compounds from an initial screen into higher mammalian models to be able to translate the data to humans and ultimately find a cure for PD. Further, this platform may also prove applicable to explore the impact of small-molecule inhibitors on other diseases. For example, the accumulation of α-synuclein contributes to the aggregation of islet amyloid polypeptide (IAPP), which has been linked to type 2 diabetes [103,104].

To test the platform further, a number of drugs were screened for their effectiveness to rescue the body bending deficit in α-synuclein-expressing worms and a reduction in the number and size of the aggregates. One of the compounds, Ambroxol, was extremely interesting, especially as it is a highly promising new treatment for PD [57,58]. In our platform, the α-synuclein aggregates were almost completely abolished in worms exposed to 3 mM Ambroxol (Figure 5a and Supplemental Figure S4). However, when tested in the mobility assay, these animals appeared to be paralyzed. It is possible that the mobility defects are a consequence of the elevated solvent percentage [88,89], which, in this case, is 3% DMSO. This was unavoidable due to the limited solubility of Ambroxol, and alternatives may be more toxic to the nematodes. Additional studies will be required to separate the response of *C. elegans* to Ambroxol and DMSO. Further, it is of interest to explore the effect of Ambroxol on α-synuclein aggregation, in terms of the aggregates themselves [105], but also to observe how the kinetics of aggregation alters in the presence of Ambroxol [106,107,108].

From our screen, we also identified other drugs that are of interest as treatments for PD. We showed that Valproic Acid reduces the number of α-synuclein aggregations, suggesting a positive effect at modulating disease progression. This is in line with previous studies demonstrating that Valproic Acid has a neuroprotective effect in nematodes [109] and in rodents [78,110]. The recovery of the mobility defect in worms that express α-synuclein in the presence of Valproic Acid also recapitulates the results observed in rats [79]. Bexarotene, Galantamine, and Tetrabenazine are interesting candidates for further research as these compounds caused a reduction in the number of α-synuclein aggregates but an increase in size, suggestive of a disruption of the aggregation process. This is striking as low doses of Bexarotene have been shown to prevent dopamine neuron degeneration in rats [81]. Further confirmation of Bexarotene as a potential drug for PD is that the mobility defect is rescued in *C. elegans* expressing α-synuclein, similar to the situation in rats [81]. Unlike Bexarotene, the mobility was not rescued in worms exposed to Galantamine or Tetrabenazine. Galantamine functions as an acetylcholinesterase inhibitor [111], and there is evidence that Galantamine, as with other cholinesterase inhibitors, is able to treat people with Parkinson’s Disease dementia [83,112]. While there are some cholinergic motor neurons in *C. elegans*, it is likely that the assay used in this platform is not sensitive enough to observe behavioral changes. Therefore, in future studies, more detailed experiments would be needed. An effect on body bending following exposure to Tetrabenazine was expected as this compound increases extracellular dopamine [113]. However, as Tetrabenazine is a VMAT2 agonist, which inhibits dopamine uptake, it is therefore not likely to affect nematode mobility [114]. Taken together, using *C. elegans* to explore the effects of compounds on two hallmarks of PD in parallel enables the drug discovery pipeline to be more streamlined and contributes to the fast identification of possible lead compounds for further mammalian studies.

## 4. Materials and Methods

### 4.1. Strains and Nematode Preparation

Strains used in this study were wild-type *N2* var. Bristol and *NL5901* (pkIs2386 (*unc-54p*::alpha-synuclein::YFP + *unc-119*^(+)^)). Nematode strains were provided by the *Caenorhabditis* Genetics Centre (CGC) and maintained on Nematode Growth Media (NGM) agar prepared according to standard protocols [115] and plates seeded with *OP50 E. coli* as a bacterial food source.

To synchronize the worm population, gravid animals were washed from plates and bleached with alkaline hypochlorite solution (4 mL of 5% sodium hypochlorite, 1 mL of 4 M sodium hydroxide, and 5 mL of dH_2_O) according to standard protocols [116]. The eggs were left to hatch overnight in M9 buffer in the absence of food at room temperature (19–20 °C). The following day, the synchronized L1 larvae could then be placed directly onto NGM.

### 4.2. Small-Molecule Modulators

The small-molecule drugs studied in this work are summarized in Table 2. They were dissolved without further purification to 100 mM in DMSO or water and subsequently diluted to 0.1, 1, and 3 mM. Betulin required sonication to fully dissolve. Stocks were made fresh for each use and then filter-sterilized before being added to molten NGM to the required concentration.

### 4.3. Treatment

Age-synchronized L1s were cultivated on standard NGM plates. When at the L4 stage, the worms were transferred to seeded NGM plates containing the drug of interest at the desired concentration with 100 µM 2′-Deoxy-5-fluorouridine (FUdR) to prevent progeny from hatching or NGM supplemented with FUdR and compounds of interest. In all cases, NGM supplemented with FUdR were considered the control plates. Plates were incubated at 20 °C until the worms were scarified for microscopy at 1 week (L4 + 7 days) or 2 weeks (L4 + 14 days) of age, as described in each experiment.

### 4.4. Thrashing Assay and Analysis

L4 worms were transferred to drug-supplemented NGM and incubated at 20 °C until the desired stage was reached. The number of body bends was quantified by placing the worms into M9 buffer (40 µL) on an unseeded NGM plate at room temperature. Videos were taken using a Leica S8aP0 binocular microscope with a Leica DMC2900 camera and the LAS v4.12 software [117]. The 90 s movies were analyzed using ImageJ v1.53 software with the wRMTrck plugin (build 110622) [49] used to quantify the number of body bends. Please see Supplemental Movie S1 to observe how body bends are counted by the plugin. The frames were normalized to that of the highest light intensity and the background was removed. Movies were set to 28 frames per second with a bend threshold of 1. Each worm was assessed for number of body bends per minute (BBPM). For the drug assessment, both *N2* (wild-type) and *NL5901* (α-synuclein-expressing worms) were assessed after 1 week of exposure (L4 + 7 days) with at least 10 worms per condition. Any worms with less than 5 body bends per minute (BBPM) were removed from the data. All the data were combined and an average calculated and plotted in GraphPad Prism v9. The error bars were calculated as the standard error of the mean (s.e.m.), which is the standard deviation divided by the square root of the number of samples.

### 4.5. Fluorescent Microscopy

Worms of the desired age were mounted on 1% agarose pads in 20 mM sodium azide. Fluorescent imaging was carried out using a Zeiss Imager.M2 microscope at 63× magnification. Images were taken using the Zeiss Zen 2012 Blue Software at 7.4 pixels/µm and representative images compiled using Adobe Photoshop 7.0. Confocal imaging of worms was undertaken on an inverted Zeiss Axio LSM700 equipped with a 488 nm diode laser and LP490 filter. Images were taken at x40 magnification, 1024 × 1024 resolution, with 4× averaging and a Z-stack slice of 1 µm. Stacks were made through the head region of the full animal to encompass all of the fluorescent signal, and then compressed to a maximum projection with Zen Blue 2011 SP3 v8.1 software at 6.4 pixels/µm and compiled in Adobe Photoshop 7.0.

### 4.6. Quantification of Plaques

To quantify the aggregates, ImageJ v1.51 was used, with no additional plugin. Each image was scaled appropriately, the background removed, and a watershed applied. Subsequently, ImageJ supplied the number of aggregates per image and the average size of aggregates in µm^2^ (calibrated units) was calculated by the ImageJ program. The data were inputted into GraphPad Prism v9 where the averages could be plotted with error bars calculated as the standard error of the mean (s.e.m.).

### 4.7. Lifespan Assay

Worms (strains *N2* and *NL5901*) were age-synchronized and placed on NGM plates containing 100 µM FUdR when at the L4 stage. The plates were maintained at 20 °C throughout the experiment and the worms transferred every 2 days during reproduction to a freshly seeded NGM plate. Worms were assessed daily for death, which was verified when they failed to respond to a gentle tap on the head with a platinum wire. Lifespan was evaluated for worms exposed to Levodopa and Ambroxol, where the same protocol was followed, but in addition to FUdR, the NGM was supplemented with the drug to a final concentration of 1 mM. In all cases, the lifespan was undertaken in 2 independent experiments and combined. Survival curves were generated in GraphPad Prism v9 and analyzed with OASIS 2.0 (Online Application for Survival analysis) [118]. Within OASIS, basic survival analysis was used to calculate the mean/medium lifespan that was assessed using the log rank test (log-rank test) where *p* < 0.0055 was considered as significant (Bonferroni corrections).

## 5. Conclusions

We further characterized the *C. elegans* PD model where the disease-causing α-synuclein is expressed in the body wall muscles, exploring the effect of the accumulation of aggregates on lifespan and mobility. We were able to use non-specialist equipment and freely available software to quantify the size and number of plaques, providing an indication as to whether drugs are able to interfere with α-synuclein aggregation, a promising disease-modifying approach for treatment of PD [36,38]. Using this information, we developed an affordable, simple, and effective assay that can be used to explore the effect of possible treatments for PD on multiple hallmarks of the disease. From the selection of compounds, Ambroxol was identified as highly promising for further studies, which is confirmed by its current status in other trials as a PD treatment. Indeed, the platform does not have to be limited to the exploration of small molecules that impact the disease progression, but could also be applied to other possible therapies including nutrition [119] and exercise [120,121]. We believe that using *C. elegans* in this drug discovery platform is invaluable for identifying promising candidate small-molecule inhibitors of PD, which can then be prioritized for validation in mammalian models.

## Figures and Tables

**Table 1:** Survey of the effect of drugs on body bending. Worms (strain *N2* and *NL5901*) were exposed to each drug for 1 week (L4 + 7 days) after which they were assessed for body bending. All drugs were tested at 0.1, 1, and 3 mM, with the exception of Ambroxol, Betulin, and Tetrabenazine, which were not tested at 3 mM (shown by an “n/a”), as the worms paralyzed immediately upon being placed in the M9 buffer. For graphical representation, see Supplemental Figure S2. All experiments were repeated once, with a minimum of 11 worms per condition. The controls (FUdR and DMSO) were run with each experiment but are combined in the table.

	*N2* (Wild-Type)			*NL5901* (α-Synuclein)		
	0.1 mM	1 mM	3 mM	0.1 mM	1 mM	3 mM
Control	32			27		
0.1% DMSO	32			37		
1% DMSO	38			37		
3% DMSO	31			22		
Ambroxol	33	34	n/a	23	21	n/a
Betulin	40	38	n/a	40	42	n/a
Bexarotene	33	41	32	33	39	38
Galantamine	22	29	32	21	17	20
Tetrabenazine	36	39	n/a	24	22	n/a
Valproic acid	40	39	46	34	41	40

**Table 2:** Small-molecule inhibitors used in the study.

Drug	Solvent	Order Number	Molecular Weight (g/mol)	Company	Disease
Ambroxol	DMSO	A9797	378.10	Sigma Aldrich	Parkinson’s
Betulin	DMSO	B9757	442.72	Sigma Aldrich	Parkinson’s
Bexarotene	DMSO	SML0282	348.48	Sigma Aldrich	Alzheimer’s
Galantamine	Water	Y0001279	368.27	Sigma Aldrich	Alzheimer’s
Levodopa	Water	PHR1271	197.19	Merck	Parkinson’s
Tetrabenazine	DMSO	T2952	317.42	Sigma Aldrich	Huntington’s
Valproic Acid	Water	PHR1061	144.21	Sigma Aldrich	Alzheimer’s Parkinson’s

## Data Availability

The data generated during this study are included in this article. Raw data can be provided upon request to the corresponding authors.

## Supplementary Materials

The following supporting information can be downloaded at: https://www.mdpi.com/article/10.3390/ph15050512/s1, Figure S1: The size and number of α-synuclein aggregates from fluorescent microscopy; Figure S2: The effect of a selection of drugs on mobility; Figure S3: The effect of Ambroxol on lifespan; Figure S4: The effect of Ambroxol on α-synuclein aggregates; Figure S5: A DMSO dose response on thrashing and α-synuclein aggregates; Table S1: Description of the mean and maximal lifespan of wild-type and worms expressing the α-synuclein following the different conditions [118,122]; Movie S1: A video to show the body bending of *C. elegans* and associated counting in ImageJ.
