# Supplementary material for: Using a Caenorhabditis elegans Parkinson’s Disease Model to Assess Disease Progression and Therapy Efficiency
Source: Pharmaceuticals (Basel). 2022 Apr 22;15(5):512. doi: 10.3390/ph15050512 (PMC9143865; doi:10.3390/ph15050512)
Supplement: Supplementary file 1 [file pharmaceuticals-15-00512-s001.zip › pharmaceuticals-1666910-supplementary.pdf]

pharmaceuticals

Supplementary Information for

Using a *Caenorhabditis elegans* Parkinson's Disease Model to Assess Disease Progression and Therapy Efficiency

Samantha Hughes [1,2,*,†], Maritza van Dop [1], Nikki Kolsters [1], David van de Klashorst [1], Anastasia Pogosova [1] and Anouk M. Rijs [3,*,†]

[1] HAN BioCentre, HAN University of Applied Sciences, Laan van Scheut 2, 6525 EM Nijmegen, The Netherlands; maritza.vandop@han.nl (M.v.D.); nikkikolsters@live.com (N.K.); david.vandeklashorst@han.nl (D.v.d.K.); anastasia.s.pogosova@gmail.com (A.P.)
[2] A-LIFE Amsterdam Institute for Life and Environment, Section Environmental Health and Toxicology, Vrije Univeristeit Amsterdam, De Boelelaan 1085, 1081 HV Amsterdam, The Netherlands
[3] Division of BioAnalytical Chemistry, AIMMS Amsterdam Institute of Molecular and Life Sciences, Vrije Univeristeit Amsterdam, De Boelelaan 1085, 1081 HV Amsterdam, The Netherlands
* Correspondence: s.hughes@vu.nl (S.H.); a.m.rijs@vu.nl (A.M.R.)
† These authors contributed equally to this work.

This file includes:
Figure S1. The size and number of α-synuclein aggregates from fluorescent microscopy.
Figure S2. The effect of a selection of drugs on mobility. Supplementary Figure 3. The effect of Ambroxol on lifespan
Figure S4. The effect of Ambroxol on α-synuclein aggregates.
Figure S5. A DMSO dose response on thrashing and α-synuclein aggregates.
Table S1. Description of the mean and maximal lifespan of wild type and worms expressing the α-synuclein following the different conditions.
Movie S1. A video to show the body bending of *C. elegans* and associated counting in ImageJ.

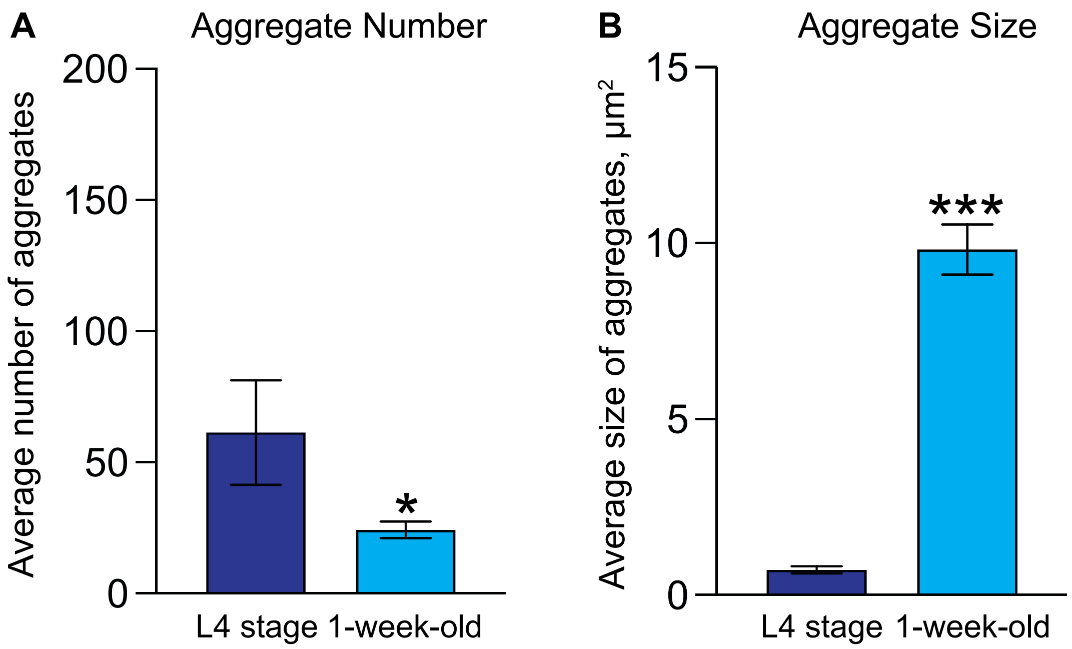

Figure S1. The size and number of α-synuclein aggregates from fluorescent microscopy. Images of *NL5901* at L4 and 1-week of age were taken with a conventional florescent microscope, Zeiss Imager.M2, and quantified using ImageJ. 2-week-old worms were not observed. **(A)** Quantification of the number of aggregates and **(B)** size in μm^2 of the aggregates were plotted in GraphPad Prism v9. The dark blue bars are L4 stage (n = 6) and the blue bars show 1-week old animals (n = 14). Graphs shown are averages with the standard error of the mean, where eat dot is the quantification from a single worm Statistical analysis is the 2-tailed 2-sample t-test, where the averages are compared to L4 stage worms. Asterisks indicate p-value, where * $p < 0.05$ and *** $p < 0.001$.

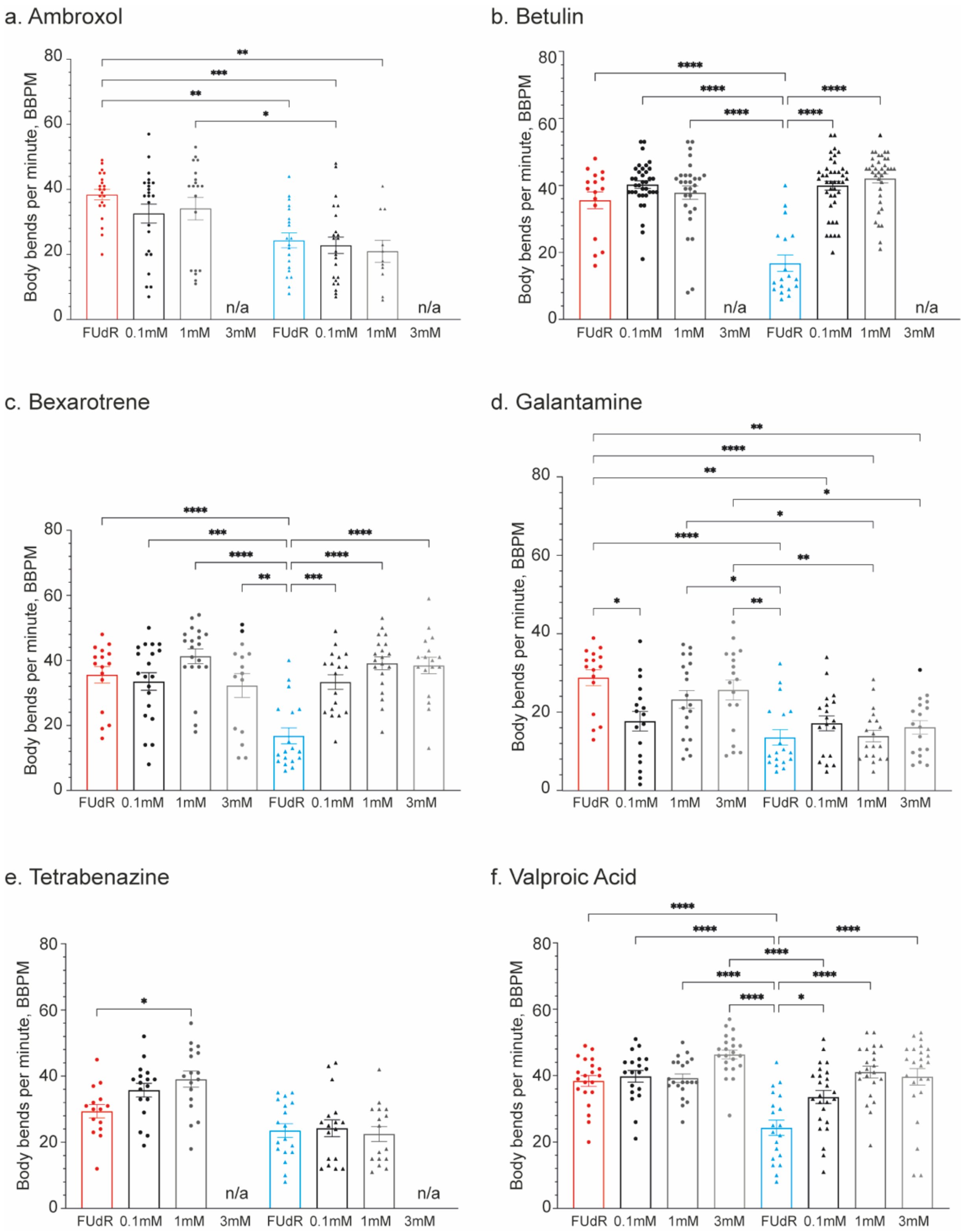

Figure S2. The effect of a selection of drugs on mobility. The average body bends per minute (BBPM) were assessed in *N2* (circles) and *NL5901* (triangles) on control (FUdR) or drug supplemented NGM at 0.1 mM, 1 mM or 3 mM. BBPM was assessed using wRMTrck in ImageJ and averages plotted with the standard error of mean in GraphPad Prism v9. The statistical tests are a one-way ANOVA where * $p < 0.05$, ** $p < 0.01$, *** $p < 0.005$ and **** $p < 0.001$. n/a indicates no worms were assessed. The blue bars show the control, FUdR; dark grey bars represent exposure to 0.1 mM for 1 week, light grey indicates 1 mM exposure and white bars are for worms exposed to 3 mM of the drug. **(a)** Exposure to Ambroxol (*N2* *n* = 23, 19, n/a; *NL5901* *n* = 23, 11, n/a); **(b)** Betulin (*N2* *n* = 39, 29, n/a; *NL5901* *n* = 41, 37, n/a); **(c)** Bexarotene (*N2* *n* = 20, 20, 15; *NL5901* *n* = 18, 20, 17); **(d)** Galantamine (*N2* *n* = 17, 19, 18; *NL5901* *n* = 19, 19, 18); **(e)** Tetrabenazine (*N2* *n* = 17, 17, n/a; *NL5901* *n* = 17, 16, n/a); **(f)** Valproic Acid (*N2* *n* = 19, 21, 22; *NL5901* *n* = 25, 22, 24).

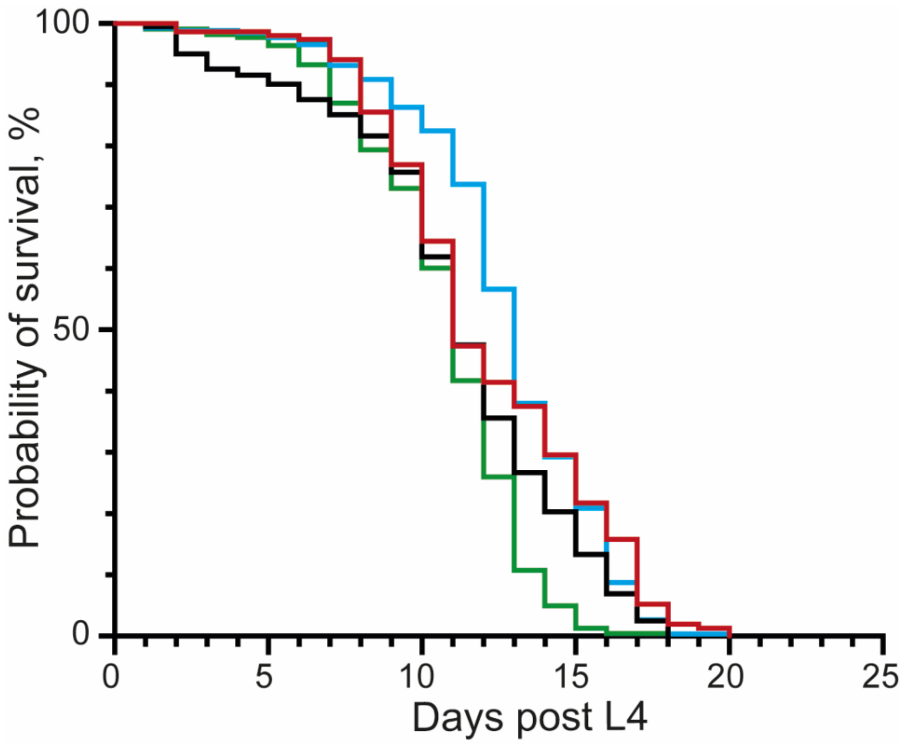

Figure S3. The effect of Ambroxol on lifespan. Worms were assessed for survival on NGM supplemented with FUdR in the presence of 1 mM Ambroxol (Wild type strain *N2*, black line; PD strain *NL5901*, green line) or 1% DMSO vehicle control (Wild type strain *N2*, red line; PD strain *NL5901*, blue line). Details of the mean and maximal lifespan are shown in Supplementary Table S1.

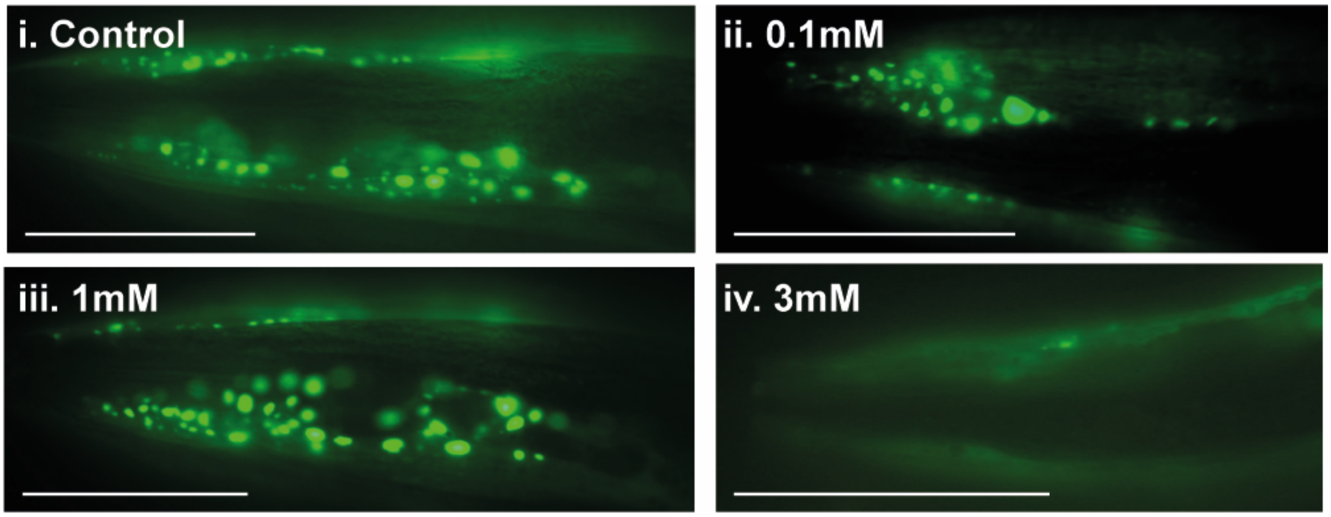

Figure S4. The effect of Ambroxol on α-synuclein aggregates. Representative Zeiss image of the head of 1-week old animals showing the α-synuclein aggregates in (**i**) control conditions, (**ii**) exposure to 0.1 mM (**iii**) exposure to 1 mM and (**iv**) 3 mM Ambroxol. Scale bar, 50 μm.

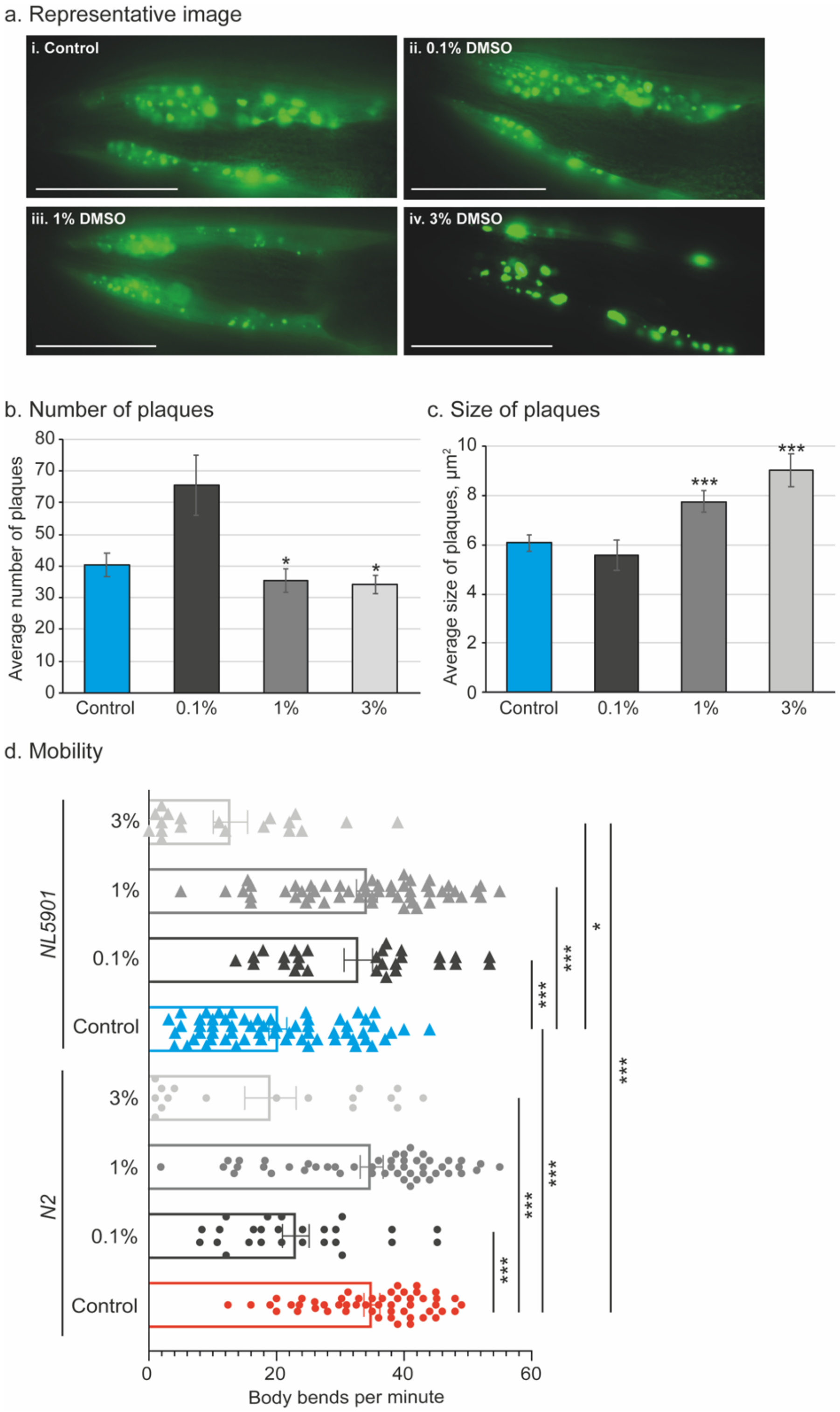

Figure S5. A DMSO dose response on thrashing and α-synuclein aggregates. **(a)** Hallmarks of the Parkinson's Disease shown by the α-synuclein aggregates. Representative Zeiss image of the head of 1-week old animal. Representative images of (**i**) Control FUdR, (**ii**) 0.1%, (**iii**) 1% and (**iv**) 3% DMSO. Scale bar, 50 μm. **(b)** Quantification of the number and **(c)** size of α-synuclein aggregates from Zeiss images. In both cases, the average is plotted with the standard error of the mean where the FUdR control (blue bars; $n = 62$) and 0.1% (black bars; $n = 26$), 1% (dark grey bars; $n = 69$) and 3% DMSO (light grey bars; $n = 45$). Exposure to DMSO at 1% and 3% results in a small but significant decrease in the number of aggregates ($p < 0.05$) but these are much greater in size ($p < 0.001$). **(d)** The average body bends per minute (BBPM) were assessed in *N2* (circles) and *NL5901* (triangles) in control (FUdR, blue bars; $n \geq 52$), 0.1% (black bars; $n \geq 26$), 1% (dark grey bars; $n \geq 49$) or 3% (light grey bars; $n \geq 17$) DMSO supplemented NGM. BBPM was assessed using wRMTrck in ImageJ and averages plotted with the standard error of mean in GraphPad Prism v9. The only statistical difference from the 2-tailed 2-sample t-test is shown, where *** indicates $p < 0.001$ and * $p < 0.05$.

Table S1. Description of the mean and maximal lifespan of wild type and worms expressing the α-synuclein following the different conditions. Lifespan was assessed in 2 independent experiments and the data combined for wild type (strain *N2*) and the worms expressing the α-synuclein (strain *NL5901*). Worms were exposed to NGM supplemented with FUdR and 1 % DMSO, 1 mM Levodopa or 1 mM Ambroxol. The data was analyzed using OASIS2 [1]. As well as the age at 50 % and 100 % mortality, the Bonferroni test was used to calculate a *p*-value, which is shown comparing all data to either *N2* or *NL5901* under control (FUdR) conditions. While we observe a small increase in survival at 50% and 100% mortality in worms expressing the α-synuclein transgene compared to wild type worms, this may be explained by a slight developmental delay [2].

Strain	Condition	*n*	Age Post L4 in Days at		Bonferroni *p*-Value to *N2* FUdR	Bonferroni *p*-Value to *NL5901* FUdR
			50% Mortality	100% Mortality		
N2	FUdR	202	10	18	-	0.0131
	DMSO 1%	152	11	20	0.0000	
	Ambroxol	202	11	18	0.0003	
	Levodopa	227	11	25	0.0929	
NL5901	FUdR	227	11	20	0.0131	-
	DMSO 1%	263	13	20		0.0000
	Ambroxol	223	11	18		0.1463
	Levodopa	240	13	20		0.0001

Movie S1. A video to show the body bending of *C. elegans* and associated counting in ImageJ. 90 second movies were made using a Leica S8aP0 binocular microscope with a Leica DMC2900 camera and the LAS v4.12 software. Movies were analysed using ImageJ v1.53 software with the wRMTrck plugin (build 110622) which was able to count the thrashes. (A) A representative 3 second clip from a movie showing a single worm in a droplet of M9 on an unseeded plate. (B) The same clip analysed using the ImageJ wRMTrck plugin. The body bend counts are shown.

References

1. Han, S.K.; Lee, D.; Lee, H.; Kim, D.; Son, H.G.; Yang, J.-S.; Lee, S.-J.V.; Kim, S. OASIS 2: Online application for survival analysis 2 with features for the analysis of maximal lifespan and healthspan in aging research. *Oncotarget* **2016**, *7*, 56147–56152.
2. Wang, Y.A., Snoek, B.L.; Sterken, M.G.; Riksen, J.A.G.; Stastna, J.J.; Kammenga, J.E.; Harvey, S.C. Genetic Background Modifies Phenotypic and Transcriptional Responses in a *C. Elegans* Model of A-Synuclein Toxicity. *BMC Genom.* **2019**, *20*, 232. https://doi.org/10.1186/s12864-019-5597-1.
